# Maternal Nutrition Affects Nitrogen Isotopic Signature in Blood Plasma of Beef Cattle Dams and Their Offspring

**DOI:** 10.3390/metabo12121249

**Published:** 2022-12-10

**Authors:** Gabriela de Pauli Meciano, Fernando José Schalch Junior, Guilherme Henrique Gebim Polizel, Arícia Christofaro Fernandes, Roberta Cavalcante Cracco, Arlindo Saran Netto, Rodrigo da Costa Gomes, Nara Regina Brandão Cônsolo, Miguel Henrique de Almeida Santana

**Affiliations:** 1 Department of Animal Science, College of Animal Science and Food Engineering—USP, Av. Duque de Caxias Norte, 225, Pirassununga 13635-900, Brazil; 2 Embrapa Beef Cattle, Av. Radio Maia, 830, Campo Grande 79106-550, Brazil

**Keywords:** fetal programming, molecular effects, prenatal nutrition, stable isotopes

## Abstract

This study evaluated the effects of gestational supplementation strategy on nitrogen isotopic signature in blood plasma of beef cows and their progeny. The study comprised 15 pregnant Nellore cows divided into three different supplementation protocols: NP) non-programmed group; PP) cows receiving protein–energy supplement in the last third of pregnancy; and FP) cows receiving protein–energy supplement throughout the gestational period. Blood plasma from cows was sampled at the beginning of gestation, in the prepartum, and postpartum periods as well as from their calves at 30 and 180 days of age, for the analysis of stable isotope ratios 15 N/14 N. At pre- and postpartum periods, cows fed PP and FP presented greater abundance of δ15 N compared to NP (*p* < 0.05) at pre- and postpartum. All three groups showed significant differences (*p* < 0.05) in the postpartum period. The δ15 N values of calves at 30 days of age differed between the NP group and PP and FP groups (*p* < 0.05), with no difference (*p* > 0.05) at 180 days of age. The different gestational supplementation strategies influenced isotopic fractionation of nutrients of cows and their calves after birth, indicating effects on nutritional metabolism and cumulative behavior on isotope abundance related to consumption during gestation.

## 1. Introduction

In tropical countries, beef cattle are usually raised in extensive systems mainly in the cow–calf phase. Therefore, changes in rainfall and temperatures along the year directly influence production and nutritional quality of forages [1]. These changes in forage supply affect cows throughout pregnancy as the animals have different nutritional needs. The middle and last thirds of the pregnancy usually coincide with the dry season in Brazil, and this phase accounts for 75% of fetal growth [2]. Nutrient deficiency during pregnancy can hinder fetal development and growth, delay progeny growth, change N metabolism and feeding efficiency, and affect body composition, reducing meat quality [3,4,5]. Changes in fetal growth and development and postnatal life through maternal nutrition are called fetal programming [6,7].

Several studies have investigated fetal programming in beef cattle and its effect on offspring phenotyping; however, very little is known about the metabolism and molecular bases of this topic. In this sense, studies on stable isotopes have helped scientists to better understand the system-wide metabolism and biology. Wilkinson et al. [8] report that studies on stable isotopes (which started around 1930) have provided insights into the metabolism of different topics, such as variety of nutritional conditions [9], biomarker of nitrogen (N) partitioning in ruminants [10,11], efficiency of N use in dairy cows [12], and prediction of feed efficiency in growing cattle [13]. These data indicate that stable isotope variations could reflect modulations of N metabolic fluxes and N metabolism.

Therefore, the use of stable isotopes to investigate fetal programming brings a better understanding of metabolism of cows and calves, and molecular knowledge on how fetal programming affects N metabolism. There is evidence that fetal programming causes variability in the isotopic N composition in animals within the same population [14], especially in ruminants [15,16]; however, information in this field is still scarce. Therefore, this study aimed to evaluate the effects of gestational supplementation strategy on N isotopic signature in blood plasma of beef cows and their progenies.

## 2. Materials and Methods

### 2.1. Experimental Design and Management

The experiment was conducted at the Department of Animal Science, College of Animal Science and Food Engineering, University of São Paulo (FZEA/USP), in Pirassununga, São Paulo State, Brazil. The study comprised 15 Nellore cows fixed-time artificially inseminated (FTAI) with semen from four breeders with known genetic values. The cows entered the breeding season and were separated into three gestational supplementary treatments, allocated in paddocks with *Brachiaria brizantha* cv. Marandu. The treatments were offered upon confirmation of pregnancy at 30 days. The three groups received mineral supplementation (0.03% of body weight) during the entire period. The treatments comprised: NP) Non-Programmed, no protein–energy supplementation during gestation; PP) Partially Programmed, protein–energy supplementation in the last third of gestation (estimated consumption of 0.35% body weight); and FP) Full Programmed, protein–energy supplementation throughout gestation (estimated consumption of 0.35% body weight). After calving, protein–energy supplement was ceased for the cows and their progenies. All groups were kept under the same sanitary and nutritional conditions.

Two days after birth calves were weighed, and all sanitary procedures were applied regarding this period. Calves were kept with their mothers in the same system and management in paddocks with *Brachiaria brizantha* cv. Marandu without private supplementation (creep feeding). More details can be found in [17].

### 2.2. Bromatological Analysis of Supplements and Pastures

Supplements offered to the cows during the gestational period were evaluated for their chemical composition, and the mineral contents are shown in Table 1. Forages were sampled by randomly collecting five areas of 1 m^2^ in each paddock, avoiding areas with feces and invasive plants. The five samples were homogenized, and a single 300-g sample was obtained. Samples to determine dry matter (DM) were oven dried by forced air ventilation at 65 °C for 72 h [18] and later ground in a 2-mm sieve for the bromatological and mineral analyses. The pasture morphological composition was determined by separating leaf material, stems, and dead material, which were dried afterward. This allowed estimating feed consumption and characterizing pasture conditions of each treatment (Table 2).

The bromatological and mineral analyses (Table 3) were conducted at the bromatology and mineral laboratory at the university. Crude protein (CP) was determined by the methodology of Silva and Queiroz [18], neutral detergent fiber (NDF) by Van Soest [20], and abundance of minerals was determined by Inductively Coupled Plasma Optical Emission Spectrometry (ICP-OES), according to Sindirações [21].

### 2.3. Processing of Forage and Supplements for the Isotopic Analysis

To evaluate isotopic fractionation of the different treatments in cows, samples of supplements offered during pregnancy (mineral and protein–energetic) were packed in sterilized plastic pots with approximately 30 g of material, transported to the laboratory and ground for the analysis of Δ15 N (15 N/14 N), under the protocol of CENA/ESALQ-USP.

The two supplement samples (mineral and protein–energetic) and picket leaves samples were ground in a 4-blade Macro Willer mill to avoid atom accumulation due to sample contamination. The grinding protocol consisted of mill aspiration at the beginning and end of each grinding procedure, followed by a jet of compressed air to remove gross remaining solids, and wash with deionized water and subsequent cleaning with 70° liquid alcohol. Afterward, the mill was dried using compressed air and the samples were ground. From the ground content, a homogeneous sample was generated in a 23-mL sterilized acrylic tube and subjected for the analysis of N isotopic fractionation (Δ15 N).

### 2.4. Blood Plasma Collection and Processing

Thirty animals, five cows per group with their respective progenies (NP, PP and FP), were selected randomly to evaluate the effect of maternal supplementation strategies on nitrogen isotopic fractionation of cows and calves. Blood samples were collected by venipuncture of the jugular vein in vacuum tubes containing EDTA (anticoagulant), at 30 and 240 days of gestation and at 30 days postpartum for the evaluation of isotopes of cows, and at 30 and 180 days of age of calves. The material was identified and placed on ice until processing in a centrifugation laboratory to obtain blood plasma (15 min at 3000 rpm and 4 °C). After pipetting the supernatant with 1000 µL pipettes in 2 mL microtubes, the plasma was kept in an ultra-freezer (−80 °C). For the isotopic analysis, microtubes were thawed and 30 µL of sample were pipetted into sterile Eppendorf tubes and shipped with ice to the Center of Nuclear Energy in Agriculture/USP (CENA) at the Stable Isotope Laboratory.

### 2.5. Isotopic Analysis of Delta 15 N (15 N/14 N)

The Δ15 N analysis to evaluate N isotopic fractionation was carried out at the CENA/USP, Piracicaba, São Paulo, Brazil. Plasma samples were pipetted to obtain 10 µL for storage in tin (Sn) capsules, inserted in the analyzer. The solids (supplements and ground leaves) were stored in the tin capsules. An IRMS spectrometer (Hydra 20-20, SerCon Co., Crewe, UK) was used, interfaced with an automatic N and C analyzer (ANCA-GSL, SerCon Co., Crewe, UK) coupled to an automatic sampler (222 XL Liquid Handler, Gilson, Madison, WI, USA). According to the equipment manual, accuracy of the analysis for natural abundance is 1.23‰ (delta per thousand 15 N) for a mass of 10 µg of N in sediments.

Inside the ANCA-GSL, the gas passed through a column containing Mg (ClO_4_)_2_ to remove water vapor, and then through a column containing Carbosorb to eliminate CO_2_. A chromatographic column (500 × 6.35 × 4 mm), filled with Carbosieve G (stationary phase) and heated to 80 °C, separated N_2_ from possible contaminants. Due to its nonpolar character, N_2_ flowed first, 80 s after the sample injection, and went to the IRMS after crossing a reduction column (CuO wires at 650 °C) that removed eluted O_2_. The O_2_ removal prevented NO formation in the ion source by the reaction between O_2_ and N_2_ that could generate a false signal mass: charge (m:z). N_2_O eluted 80 s after N_2_ passing through the oven containing CuO, reducing to N_2_. The IRMS Hydra 20-20 has three collectors that integrate the ion streams of m:z 28, 29, and 30. The atoms of 14 N and 15 N contained in N_2_ form molecules 14N14N, 14N15N, and 15N15N, written as 28N2, 29N2 and 30N2. Separation of these N_2_ molecules after ionization and their quantification in IRMS allow calculating the contribution of the source marked with 15 N to the total amount of gas produced [22].

The atmospheric air (78% by volume of N2 and 0.3663% in atoms of 15 N) was used as N_2_ standard, considering density of N_2_ equals to 1.25 µg × µL^−1^ with an analytical error of 0.2‰ (per thousand) through the dimensionless expression:

𝛿15 N = [(𝑅_sample_ − 𝑅_standard_)/𝑅_standard_] × 1000

where: 𝑅_sample_ is the isotopic ratio (15 N/14 N) measured in the sample and 𝑅_standard_ is the same ratio as a standard, in this case, atmospheric air. As the numerical values of the differences between the isotopic ratios (r) are small, it is usual to multiply the expression by 1000, obtaining the terminology in delta per thousand (δ‰).

### 2.6. Statistical Analysis

The effects of treatments (NP, PP, and FP) and time on traits were evaluated by the analysis of variance. The residues were tested for normality utilizing Shapiro–Wilk test. The age of cows and paternity were used in the linear model. The results with a significant difference (*p* < 0.05) had the means compared by the Tukey test at 5% level of significance. All statistical analyses were performed using the GLM procedure from SAS 9.3 statistical package (SAS Institute Inc., Carrey, NC, USA, 2011).

## 3. Results

### 3.1. Isotopic Evaluation of Forages and Supplements

The pastures where cows were kept until calving and where offspring remained after birth showed negative 15 N isotope (ẟ15 N), indicating that pastures were richer in 14 N isotope than in heavy isotope 15 N. Pre-calving pasture of PP and FP treatments had ẟ15 N of −1.39‰, while pre-calving pasture had ẟ15 N of −3.07‰ in the NP treatment, the lowest abundance presented. The pastures where all animals remained from birth to weaning had ẟ15 N of −2.93‰. Mineral supplement offered to all treatments had ẟ15 N of 1.89‰, while the protein–energy diet provided only to PP and FP treatments had ẟ15 N equals to 0.55‰.

### 3.2. Isotopic Evaluation of Blood Plasma of Cows

In the prepartum period of cows, PP and FP treatments had similar values of ẟ15 N, differing only from the NP group (Table 1). At the beginning of pregnancy, cows showed similar values for concentration and abundance of ẟ15 N. The 15 N isotope concentration showed a significant difference (*p* < 0.05) only between the periods in the NP treatment (*p* = 0.037), with a trend for a difference between the treatments in the prepartum period (*p* = 0.064). Abundance of ẟ15 N differed between treatments in the prepartum (*p* < 0.001) and postpartum (*p* < 0.001) periods. In the prepartum period, FP and PP treatments differed from NP, while all treatments differed from each other in postpartum. For the periods, all treatments showed differences, indicating a different abundance in each pregnancy stage (*p* < 0.05; Table 4).

### 3.3. Isotopic Evaluation of Blood Plasma from Calves

In calves, two different periods were evaluated at 30 and 180 days of age. Concentration showed no difference in any interaction (*p* > 0.05). The ẟ15 N values showed a difference between the treatments during 30 days of age (*p* < 0.01) in which PP and FP had higher values than NP.

However, the same was not observed at 180 days when all treatments displayed the same abundance (*p* = 0.878). For the ẟ15 N analysis between the periods, only FP showed difference in isotope abundance in blood concentration (*p* = 0.023; Table 5). At 30 days of age (postpartum period of cows), calves showed an enrichment (Δ15 N) of ẟ15 N of 0.86‰ in NP and FP treatments and 1.19‰ in PP in relation to their cows, where Δ15 N = ẟ15 Nprogeny—ẟ15 N 8 cow.

## 4. Discussion

To the best of our knowledge and based on a literature review, this is the first study on stable isotopes in cattle submitted to different fetal programming strategies. The data presented here suggest that diet variation of ẟ15 N reflects modulations of N metabolic fluxes and N metabolism in cows and their progeny.

In ruminants, stable isotopes in fluids or tissues vary according to the food and water ingested and the gases inhaled, and are also influenced by environmental conditions and the physiological phase of the animals [16]. Therefore, similarity between PP and FP could be attributed to the protein–energy supplementation, as both groups consumed supplements for at least 90 days. The NP treatment showed a slight enrichment between the beginning and the end of pregnancy (prepartum), due to the positive isotopic abundance (ẟ15 N of 1.89‰) in the mineral supplementation, as the pasture in this treatment was poor in ẟ15 N. The FP treatment was richer than the PP between the beginning and the end of pregnancy, possibly due to the longer supply of protein–energy supplementation, since cows fed FP received supplementation for nine months, while animals fed PP received the supplement for only three months.

In the postpartum period (30 days after birth), cows of all treatments were consuming the same diet since partum; however, the slight differences on ẟ15 N between treatments could be explained by a cumulative effect on the abundance of isotopes related to consumption during the gestation. Cantalapiedra-Hijar et al. [23] report that plasma isotopic turnover takes five months on average in Charolais bulls. However, according to Jenkins et al. [24], isotopic signature in the plasma reflects the last 7–10 days; therefore, our data are from two animal categories (cows and calves), supporting that this time interval has a wider range specific for the species.

The turnover time of five months for proteins in the plasma [23] is also useful to explain the difference between treatments in calves at only 30 days old. At this time, isotopic abundance in the plasma still reflects the abundance of fetal life, since feed uptake of offspring reflects the maternal habitat, and progenies are dependent and need suckling. The progeny–mother relationship showed that 𝛿15 N in offspring tissues simulated a progeny–mother relationship, closely related to newborns that feed almost exclusively on cow milk [25,26,27,28].

In addition, differences in calves’ isotopic abundance at 30 days of age might also be due to the greater use of N in body tissues of PP and FP calves and consequently lower N excretion. This suggests that calves from cows fed with supplement during the gestation period show a more efficient metabolism of protein and N to improve N retention. Gannes et al. [29] explain that N stable isotopes may provide an indicator of N and protein balance that can be used on free-ranging animals, since animals excrete N enriched in 14 N, resulting in an enriched tissue of animals with 15 N thus increasing the 15 N/14 N ratio. The authors also emphasize that the ration 15 N/14 N allows estimating the N balance and the physiological state of the animal [29]. This is in line with our hypothesis that improved cow nutrition promotes greater fetus hyperplasia, improving efficiency of N retention in the muscle fiber, which can improve calf performance, muscle mass and fat deposition. Similarly, other authors have reported greater muscle mass development in calves from cows that received improved diets during gestation, which might boost carcass traits, fat deposition, and subprimal yield [30,31,32].

Cantalapiedra-Hijar et al. [33] reported correlations between feed efficiency in beef cattle and ẟ15 N in blood and tissues. The same authors suggested that the assimilation efficiency of dietary N into animal proteins could influence variations in ẟ15 N [33]. The improvement of cow nutrition during pregnancy might improve feed efficiency in calves [34], as the animals could be using more efficiently the dietary protein and metabolizable protein; however, further studies are needed to confirm this hypothesis. This observation may have a close relationship with N fluxes from cows to calves, as our data showed, where calves from supplemented cows at 30 days of age presented greater plasma ẟ15 N. Nevertheless, further studies that include metabolomics data to better understand N metabolism and protein retention are needed.

The ẟ15 N values in plasma in all treatments were higher in newborn calves with 30 days of life than in their respective mothers during the postpartum period, indicating fetal enrichment and corroborating findings in the literature. Jenkins et al. [24] reported offspring plasma with an average ẟ15 N of 0.9 ± 0.8‰ above the maternal plasma. Barboza and Parker [35] suggest that offspring enrichment in ẟ15 N in relation to the cows is due to the fetal protein origin from maternal reserves, and not directly from the diet consumed by cows. These results corroborate our findings on isotopic evaluations of calves at 30 days of age.

## 5. Conclusions

Fetal programming strategies of Nellore cows promote differences on stable N isotopes signature in blood plasma of cows and their calves, which are indicative of an effect of protein-supplement supply and cumulative behavior on isotope abundance related to consumption during gestation.

## Figures and Tables

**Table 1:** Contents of ingredients and nutrients of supplement for cows.

Ingredients	Mineral Supplement	Energetic–Proteic Supplement
Ground corn (%) Soybean meal (%) Dicalcium phosphate (%) Urea 45% (%) Salt (%) Minerthal 160 MD (%) * Total digestible nutrients (%) Crude protein (%) Non-protein nitrogen (%) Acid detergent fiber (%) Neutral detergent fiber (%) Fat (%) Calcium (g/kg) Phosphate (g/kg)	35 - 10 - 30 25 26.76 2.79 - 1.25 4.29 1.26 74.11 59.38	60 30 - 2.5 5 2.5 67.55 24.78 7.03 4.76 11.24 2.61 6.2 7.24

* Guarantee levels (25 kg): calcium, 200–230 g; cobalt, 160 mg; copper, 2700 mg; sulfur, 60 g; fluorine, 1600 mg; phosphor, 160 g; iodine, 135 mg; manganese, 2700 mg; selenium, 80 mg; zinc, 8100 mg; sodium monensin, 4000 mg [19].

**Table 2:** Estimation of forage consumption by cows during the gestational period (mean ± standard deviation).

Forage Availability	Diets		
	NP	PP	FP
Pasture Availability (kg DM/ha)	3476.24 ± 1594.40	4597.35 ± 1189.80	5578.03 ± 2049.37
Leaf Availability (kg DM/ha)	573.59 ± 340.56	569.13 ± 485.76	727.49 ± 643.30
Thatch Availability (kg DM/ha)	562.46 ± 396.97	799.05 ± 545.80	1347.44 ± 1038.42
Dead material availability (kg DM/ha)	2340.07 ± 1275.00	3229.43 ± 973.34	3503.07 ± 1410.43
Stocking Rate (AU/ha)	2.19 ± 1.02	1.74 ± 0.56	2.26 ± 0.99
Leaf supply for animal unit (KG/DM)	316.64 ± 216.96	359.93 ± 245.10	366.59 ± 306.58

NP: Non-Programmed, no protein–energy supplementation during pregnancy; PP: Partially Programmed, protein–energy supplementation in the final third of pregnancy (estimated consumption of 0.35% body weight); and FP: Full Programmed, protein–energy supplementation throughout pregnancy (estimated consumption of 0.35% body weight).

**Table 3:** Chemical composition of pastures consumed by cows in the different groups (mean ± standard deviation).

Forage Nutrientes (%)		Diets	
	NP	PP	FP
Crude protein	7.38 ± 1.72	7.82 ± 2.28	7.40 ± 2.30
Total digestible nutrients	63.07 ± 1.45	64.10 ± 2.33	61.43 ± 2.12
Neutral detergent fiber	59.03 ± 3.67	61.43 ± 5.05	58.49 ± 4.11
Calcium	0.38 ± 0.11	0.35 ± 0.05	0.39 ± 0.08
Phosphor	0.19 ± 0.03	0.19 ± 0.03	0.17 ± 0.03

NP: Non-Programmed, no protein–energy supplementation during pregnancy; PP: Partially Programmed, protein–energy supplementation in the final third of pregnancy (estimated consumption of 0.35% body weight); and FP: Full Programmed, protein–energy supplementation throughout pregnancy (estimated consumption of 0.35% body weight).

**Table 4:** Plasma isotope signature of cows from different nutritional strategies and periods.

Traits	Period	Diets			*p*-Value
		NP	PP	FP	
Abundance	Initial	2.002 ± 0.086 ^A^	2.107 ± 0.151 ^A^	2.160 ± 0.078 ^A^	0.341
	Pre	2.412 ± 0.104 ^B a^	3.390 ± 0.135 ^B b^	3.796 ± 0.157 ^B b^	<0.001
	Post	2.156 ± 0.073 ^A B a^	2.722 ± 0.087 ^C b^	3.094 ± 0.090 ^C c^	<0.001
	*p*-value	0.033	<0.001	<0.001	
Concentration	Initial	1.204 ± 0.034 ^A B^	1.192 ± 0.027	1.190 ± 0.073	0.986
	Pre	1.120 ± 0.022 ^A^	1.270 ± 0.080	1.246 ± 0.060	0.064
	Post	1.254 ± 0.038 ^B^	1.280 ± 0.049	1.338 ± 0.056	0.207
	*p*-value	0.037	0.518	0.275	

NP: Non-Programmed, no protein–energy supplementation during pregnancy; PP: Partially Programmed, protein–energy supplementation in the final third of pregnancy (estimated consumption of 0.35% body weight); and FP: Full Programmed, protein–energy supplementation throughout pregnancy (estimated consumption of 0.35% body weight). Superscript uppercase letters (^A, B, C^) represent significant contrasts between periods, while superscript lowercase letters (^a, b, c^) represent significant contrasts between treatments.

**Table 5:** Plasma isotope signature of calves from different treatments and periods.

Traits	Age	Diets			*p*-Value
		NP	PP	FP	
Abundance	30 days	3.022 ± 0.149 ^a^	3.910 ± 0.095 ^b^	3.954 ± 0.150 ^b^	0.002
	180 days	3.028 ± 0.228	3.385 ± 0.221	3.134 ± 0.223	0.878
	*p*-value	1.000	0.368	0.023	
Concentration	30 days	1.082 ± 0.048	1.105 ± 0.022	1.180 ± 0.042	0.479
	180 days	1.090 ± 0.022	1.137 ± 0.049	1.178 ± 0.025	0.386
	*p*-value	1.000	0.991	1.000	

NP: Non-Programmed, no protein–energy supplementation during pregnancy; PP: Partially Programmed, protein–energy supplementation in the final third of pregnancy (estimated consumption of 0.35% body weight); and FP: Full Programmed, protein–energy supplementation throughout pregnancy (estimated consumption of 0.35% body weight). Superscript lowercase letters (a, b) represent significant contrasts between treatments.

## Data Availability

Data will be available up on request due to privacy or ethical restrictions.
